# Supplementary material for: A chiral inverse Faraday effect mediated by an inversely designed plasmonic antenna
Source: Nanophotonics. 2023 May 15;12(12):2115–20. doi: 10.1515/nanoph-2022-0772 (PMC11501613; doi:10.1515/nanoph-2022-0772)
Supplement: Supplementary file 1 — Supplementary Material Details [file j_nanoph-2022-0772_suppl_001.docx]

Supporting information

**A Chiral Inverse Faraday Effect Mediated by an Inversely Designed Plasmonic Antenna**

Ye Mou, Xingyu Yang, Bruno Gallas, and Mathieu Mivelle*

Sorbonne Université, CNRS, Institut des NanoSciences de Paris, INSP, F-75005 Paris, France

*Corresponding author: [mathieu.mivelle@sorbonne-universite.fr](mailto:mathieu.mivelle@sorbonne-universite.fr)

A list of the main content:

Simulation parameters

Supporting figures S1 to S8

**Simulation parameters:**

The simulations carried out in this study were done by the finite difference time domain (FDTD) method performed on the commercial software Lumerical from Ansys. This method solves Maxwell's equations in space and time using a finite difference technique. Indeed, the FDTD method solves these equations on a discrete spatial and temporal grid. The dimensions of the 3D computational window for the simulations were 750x750x900 nm^3^. The boundary conditions of this window are made of a perfectly matched layer (PML), avoiding any parasitic reflection inside the calculation window. Several meshes are used for the discretization of the computational space, a coarser non-uniform mesh of 4 to 16 nm for the external unstructured parts, of the simulation, a finer mesh of 4 nm for a central nanostructured part of 288x288x36 nm^3^ in X, Y, and Z, respectively containing the nanostructures, and an even finer mesh of 1 nm for the part where the drift currents and magnetic field are calculated of 140x140x32 nm^3^ in X, Y, and Z (Figure S1C). The choice of this mesh size for the central part is chosen because convergence in the amplitude of the magnetic field is observed starting from this mesh size (Figure S8). The excitation of the nanostructures is performed by a pulsed plane wave of duration 5.3 fs spectrally centered at a wavelength of 800 nm. The peak power of the pulse is 10^12^ W/cm^2^, which corresponds to an energy slightly lower than 4.8 pJ applied to the plasmonic nanostructures (energy density of 5.3 mJ/cm^2^). The convergence of the simulation was obtained when the energy inside the calculation window was lower than 10^-5^ of the initial injected energy. The textbook values of Johnson and Christy were used for the gold properties in these simulations.

**Supporting figures:**


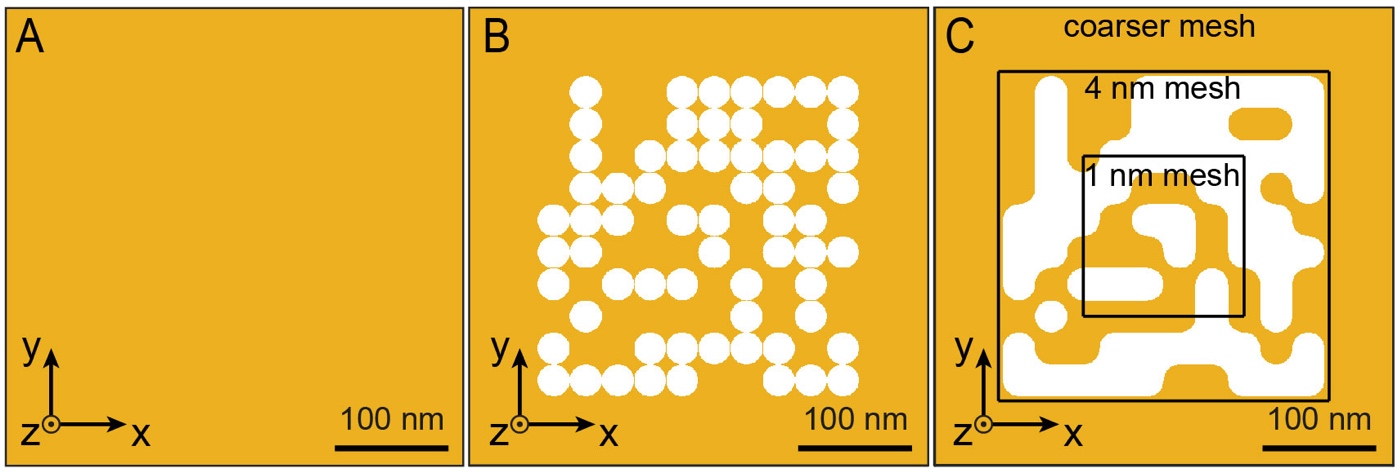


**Figure S1:** Construction of the elements constituting each generation of the genetic algorithm. Inside A) a uniform gold layer of 30 nm thickness, holes are made according to a binary matrix playing the role of the DNA in the evolutionary process. C) The obtained structure is then smoothed to avoid all the roughnesses not experimentally feasible and generating non-physical effects. The different mesh areas are shown in C.

**Figure S2:** Evolutionary process. Evolution during the different generations of the optimization function consisting in maximizing the difference **B**_RCP_-abs(**B**_LCP_), with **B**_RCP_ and **B**_LCP_ the **B**-fields created by a right or left circular polarization, respectively. Each generation consists of 200 structures. The optimized structure appears after 76 generations.


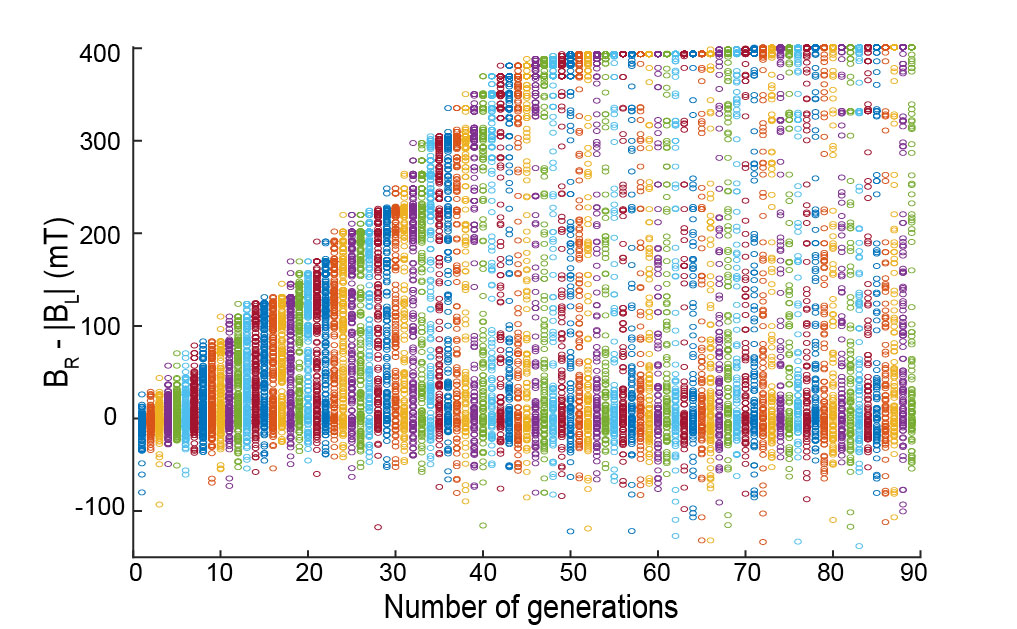


**Figure S3:** Optical responses in an XY plane. A) and B) Spatial distributions of the opti
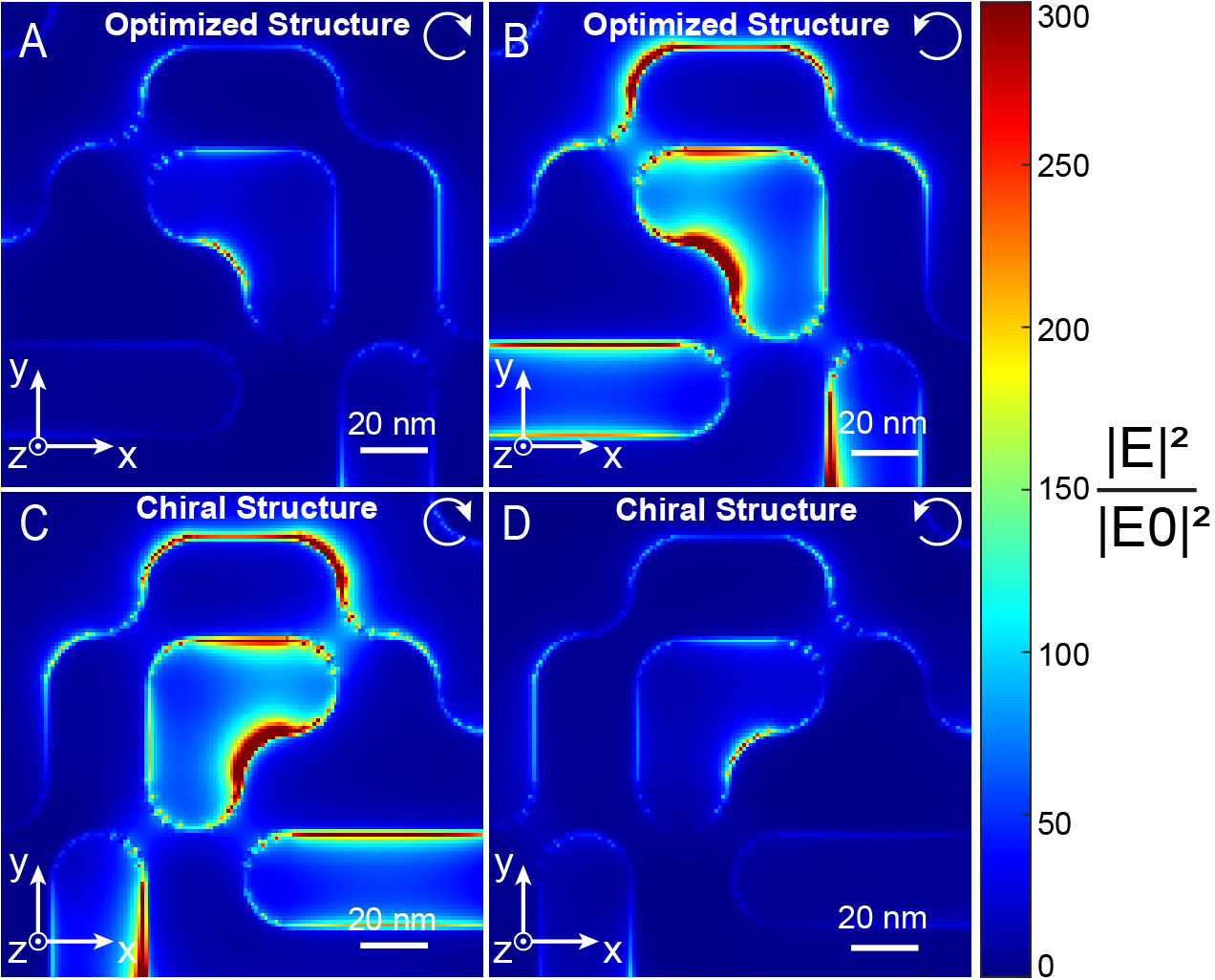
cal electric intensity enhancement at the surface of the optimized structure (2 nm below the upper edge of the gold layer) for the left and right circular polarizations of excitation, respectively. C) and D) Spatial distributions of the optical electric intensity enhancement at the surface of the mirror structure for the left and right circular polarizations of excitation, respectively. The white arrows indicate the incoming polarizations.

**Figure S4:** Distribution of drift currents in an XY plane. A) and B) Spatial distributions of drift currents at the surface (2 nm below the upper edge of the gold layer) of the optimized structure for the left and right circular polarizations of excitation, respectively. C) and D) Spatial distributions of drift currents at the surface of the mirror structure for the left and right circular
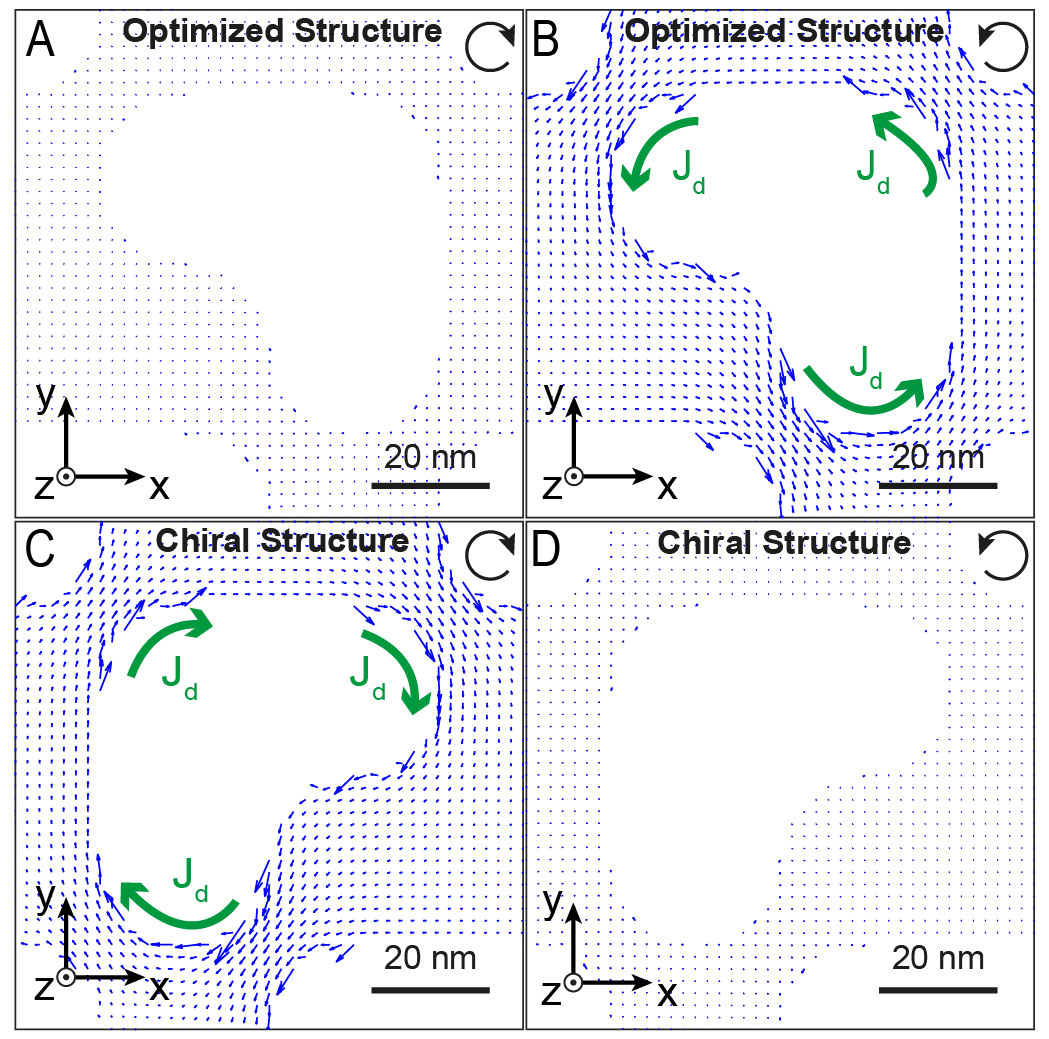
 polarizations of excitation, respectively. The black arrows indicate the incoming polarizations.

**Figure S5:** Vectorial magnetic field components. A) Schematic, in an XY plane, of the GA-optimized structure. B) and C) Spatial distributions of the **B**-field along X (**B_x_**) and the **B**-field along Y (**B_y_**) generated at the Z-center of the structure shown in A) for the right circular polarizations
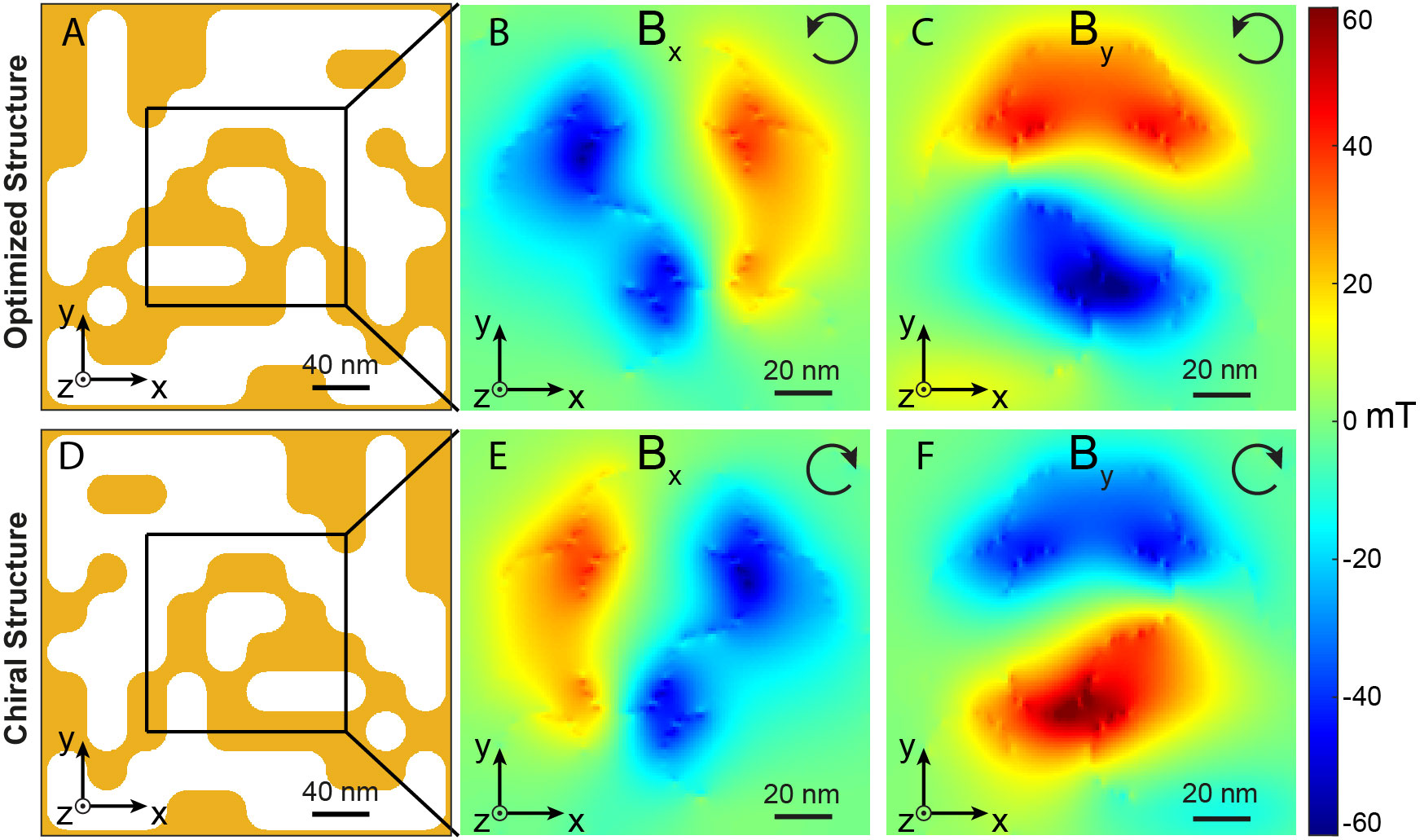
 of excitation. D) Schematic, in an XY plane, of the mirror structure. E) and F) Spatial distributions of **B_x_** and **B_y_** generated at the Z-center of the mirror structure shown in D) for the left circular polarizations of excitation. The black arrows indicate the incoming polarizations.


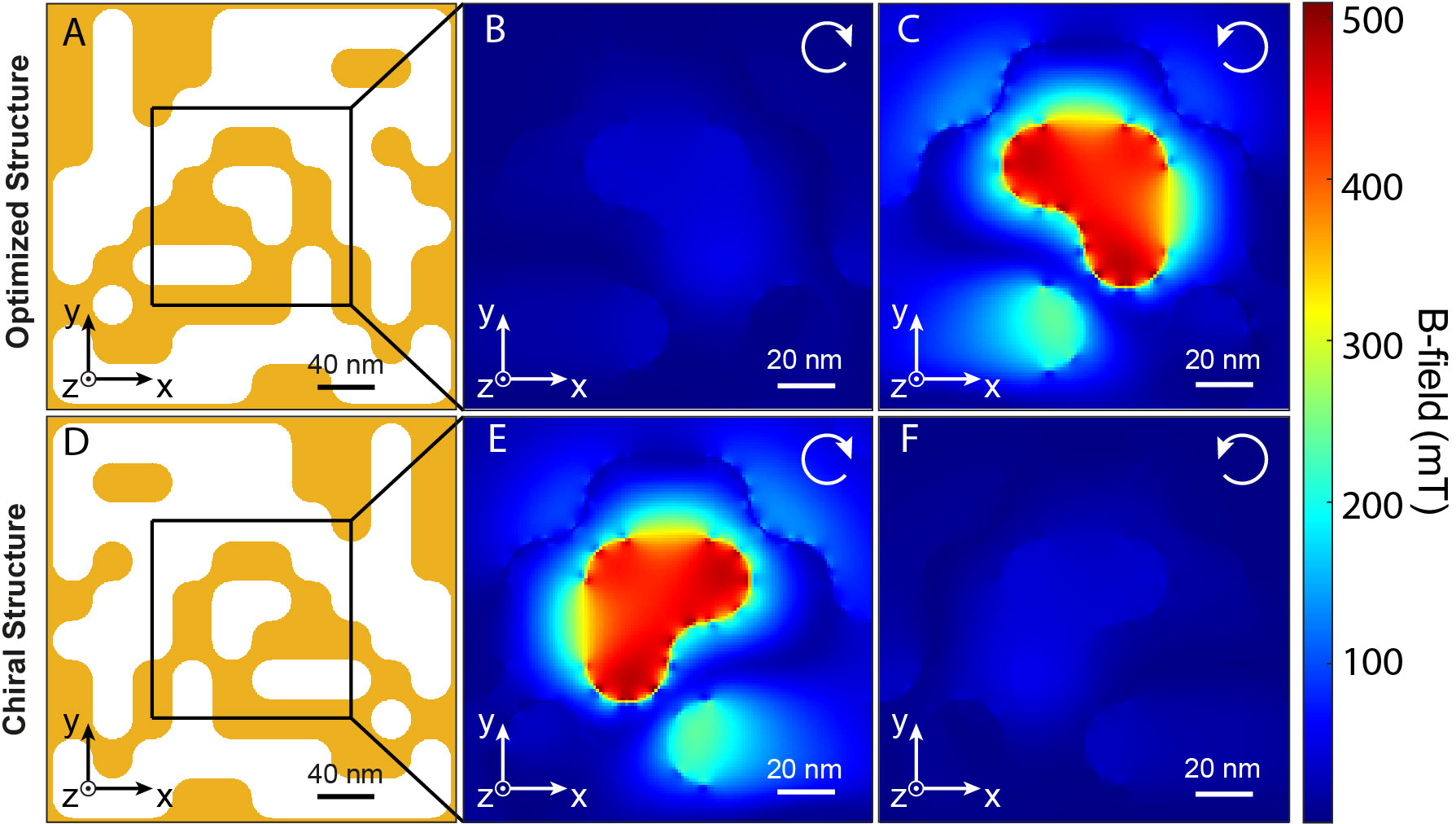
**Figure S6:** Amplitude of magnetic fields. A) Schematic, in an XY plane, of the GA-optimized structure. B) and C) Spatial distributions of the **B**-field amplitude at the Z-center of the structure shown in A) for the left and right circular polarizations of excitation, respectively. D) Schematic, in an XY plane, of the mirror structure. E) and F) Spatial distributions of **B**-field amplitude at the Z-center of the mirror structure shown in D) for the left and right circular polarizations of excitation, respectively.The white arrows indicate the incoming polarizations.


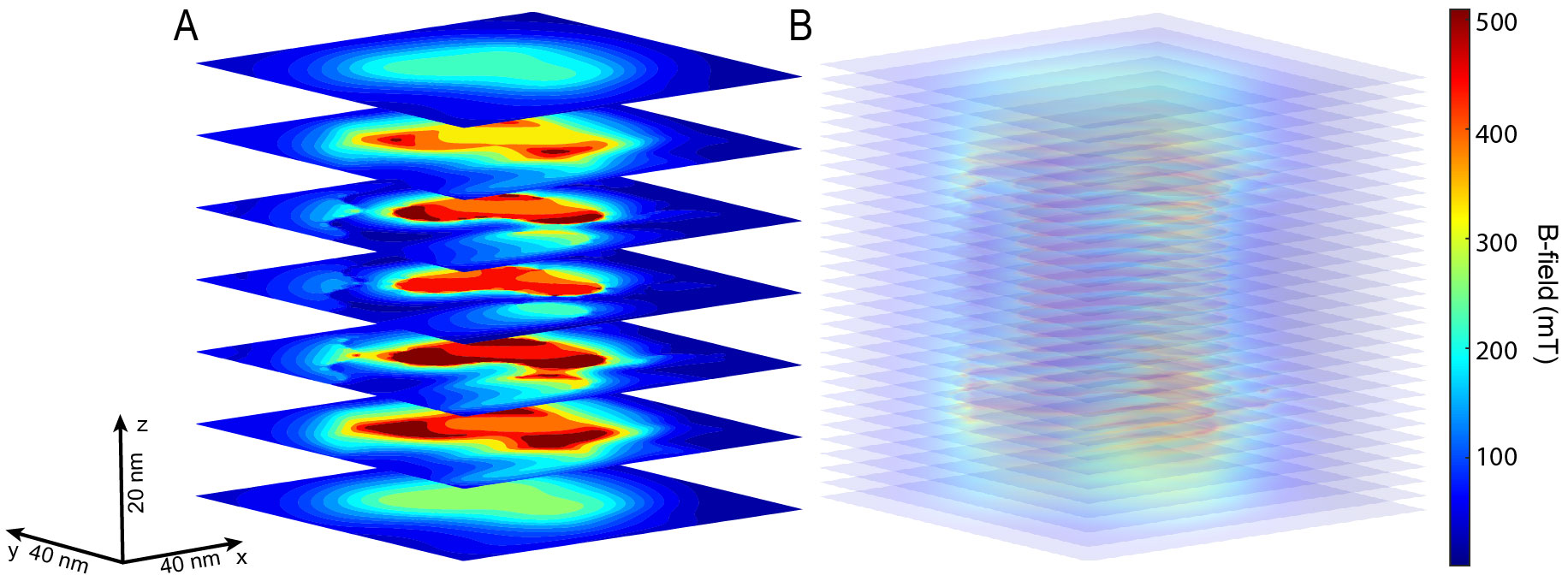


**Figure S7:** Amplitude of magnetic fields in 3D. XY amplitude distributions of the magnetic field B in different Z planes from -15 nm below the gold layer to +15 nm above it in steps of A) 10 nm and B) 2 nm. The distributions in figure B) are partially transparent in order to obtain a 3D representation of this magnetic field distribution.


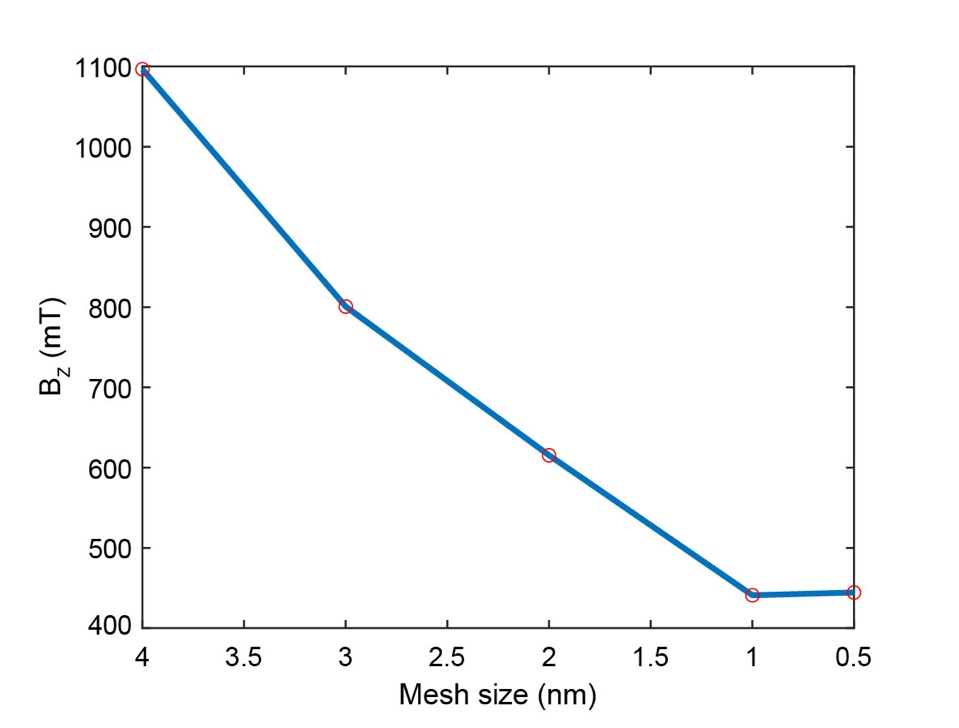


**Figure S8:** Amplitude of the magnetic field oriented along Z and generated by the optimized nanostructure for excitation by a right circular polarization and for different mesh sizes of the central zone. A convergence is observed from a mesh size of 1 nm, justifying the choice of the latter in the central zone of 140x140x32 nm^3^
